# Mechanism of Estradiol-Induced Block of Voltage-Gated K^+^ Currents in Rat Medial Preoptic Neurons

**DOI:** 10.1371/journal.pone.0020213

**Published:** 2011-05-20

**Authors:** Michael Druzin, Evgenya Malinina, Ola Grimsholm, Staffan Johansson

**Affiliations:** Department of Integrative Medical Biology, Section for Physiology, Umeå University, Umeå, Sweden; McGill University, Canada

## Abstract

The present study was conducted to characterize possible rapid effects of 17-β-estradiol on voltage-gated K^+^ channels in preoptic neurons and, in particular, to identify the mechanisms by which 17-β-estradiol affects the K^+^ channels. Whole-cell currents from dissociated rat preoptic neurons were studied by perforated-patch recording. 17-β-estradiol rapidly (within seconds) and reversibly reduced the K^+^ currents, showing an EC_50_ value of 9.7 µM. The effect was slightly voltage dependent, but independent of external Ca^2+^, and not sensitive to an estrogen-receptor blocker. Although 17-α-estradiol also significantly reduced the K^+^ currents, membrane-impermeant forms of estradiol did not reduce the K^+^ currents and other estrogens, testosterone and cholesterol were considerably less effective. The reduction induced by estradiol was overlapping with that of the K_V_-2-channel blocker r-stromatoxin-1. The time course of K^+^ current in 17-β-estradiol, with a time-dependent inhibition and a slight dependence on external K^+^, suggested an open-channel block mechanism. The properties of block were predicted from a computational model where 17-β-estradiol binds to open K^+^ channels. It was concluded that 17-β-estradiol rapidly reduces voltage-gated K^+^ currents in a way consistent with an open-channel block mechanism. This suggests a new mechanism for steroid action on ion channels.

## Introduction

Sex steroids and their metabolites influence nervous function. Besides playing important roles in the regulation of sexual behaviour [1], they also affect differentiation of the nervous system, mood and emotional behaviour, responses to stress and cognitive functions [2]–[5]. Estradiol, although being a female sex steroid, is also present in the male brain, where it causes the differentiation towards a male sexual pattern [1] and rapidly affects sexual behaviour [6], [7]. Estradiol can be synthesized de novo from cholesterol in the brain [8] and testosterone is converted to estradiol by the cytochrome P450 aromatase, which is concentrated in areas involved in reproductive control such as the preoptic area [9], but is also present in the hippocampus [8]. Aromatase activity and estradiol production can be quickly (within 5–10 min) regulated by e. g. agonists of glutamate receptors [8], [10]. Thus, the local estradiol concentration in the hippocampus may significantly exceed that found in plasma [8]. In areas controlling sexual behaviour, such as the preoptic area, the local estradiol concentration is likely to reach even higher levels, which are toxic to other organs [1], [10]. Therefore, estradiol has the potential of being a rapidly acting physiological regulator of neuronal signalling.

To understand how estradiol affects neuronal function to influence sexual behaviour and cognitive functions such as memory formation, and to develop drugs affecting these processes, information on the molecular mechanisms of action is needed. However, the mechanisms of estradiol action on nervous function are to a large extent unknown. The rapid effects on male sexual behaviour suggest that besides “classical” mechanisms via intracellular estradiol receptors and gene transcription, non-transcriptional effects at the membrane level may be involved [6], [7]. A number of electrophysiological studies have demonstrated rapid effects of estradiol in several parts of the central nervous system. Thus within seconds or minutes of application, 17-β-estradiol may alter firing rates in the preoptic area [11] and potentiate excitatory postsynaptic potentials in the hippocampus [12]. Several studies show that some of the rapid effects are mediated by K^+^ channels. Thus, Ca^2+^-dependent K^+^ channels in non-neuronal cells [13] as well as in neurons [14] are affected by estradiol.

In the present study we aimed at clarifying the acute action of estradiol on voltage-gated K^+^ currents in neurons from the medial preoptic nucleus (MPN) of young male rats. The intention was to provide insights into the mechanisms of a possible block by studying K^+^ currents under voltage-clamp conditions. Our results show that in micromolar concentration estradiol rapidly (within seconds) and reversibly reduces voltage-gated K^+^ currents. The properties of the estradiol effect suggested an action on open K^+^ channels from the inside of the membrane. A quantitative description of the voltage-gated K^+^ currents was made and used to model the interaction of estradiol with K^+^ channels. A model with an open-channel block mechanism explained the experimentally observed effects of estradiol. We conclude that estradiol reduces delayed rectifier K^+^ channels, most likely from the inside of the membrane, in a way consistent with an open-channel block mechanism.

## Materials and Methods

### Ethics statement

Ethical approval of the procedures described was given by the regional ethics committee for animal research (“Umeå djurförsöksetiska nämnd” at the Court of Appeal for Northern Norrland in Umeå; No A13-08). All efforts were made to ameliorate suffering of animals.

### Animals

Male Sprague-Dawley rats weighing 60–100 g were used for the experiments. Animals were maintained under controlled light/dark cycle (12/12 h) and temperature (22±2°C) with free access to food and water.

### Cell preparation

The method used has been previously described [15]. In short, the animals were killed by decapitation without anaesthetics. The brain was quickly removed and placed in preoxygenated ice-cold incubation solution. The meninges were removed, a block of tissue including the anterior hypothalamus and preoptic area was cut out, and slices, 300 µm thick, were cut using a vibroslicer (Campden instruments, Leicestershire, UK). The prepared slices were incubated for 1.5–2 hours in incubation solution (see below) at 27–28°C.

After the incubation period, the slices were transferred to a plastic dish and neurons were mechanically dissociated by moving the tip of a vibrating glass rod towards the MPN [16]. No enzymes were used. Dissociated cells were allowed to settle at the bottom of the dish for 30 minutes.

### Electrophysiological recordings

Whole-cell currents were recorded using the amphotericin-B perforated-patch technique [17]. Borosilicate glass pipettes (Harvard apparatus, Kent, UK) were used. The pipette tips were filled by immersion in standard intracellular solution and subsequent back-filling with intracellular solution supplemented with amphotericin-B. When immersed in standard extracellular solution, the pipette resistance was 2.5–3.5 MΩ. Series resistance compensation up to 90% was used when quantitatively important, e.g., to establish concentration response curves and voltage dependence. Due to a decay of capacitative transients that did not always follow a mono-exponential time course, some recordings (e.g. test for Ca^2+^ dependence and pharmacological manipulation) were made without series resistance compensation, to improve stability when the effect of series resistance was not critical. In all cases, the stability of series resistance was evaluated repeatedly from the time course of capacitative transients. Slow changes in series resistance less than about 20% were accepted. The liquid-junction potential was about 14 mV and has been subtracted in all potential values given [15].

The recordings were made using an Axopatch 200A amplifier, a Digidata 1200 interface and pClamp software (versions 8–9; all from Axon instruments, Foster city, USA). The leak currents and capacitative currents were subtracted using scaled current responses to negative potential steps. The finding of linear I-V relations for currents evoked in MPN neurons in response to voltage steps in the range -40 to -140 mV [18] justified this procedure.

The extracellular solution, without or with estradiol or other test substances, was applied by a gravity-fed fast perfusion system with a four- or eight-barrelled pipette positioned 100–200 µm from the cell under study. All experiments were carried out at room temperature, 21–23°C.

### Data analysis

Concentration-response curves were generated by fitting the logistic (Hill) equation:
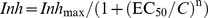
(1)to the data (raw data as well as computed currents), where *Inh* is percent inhibition with subscript “max” denoting maximum inhibition, *C* denoting concentration of estradiol, EC_50_ the half-saturating concentration and *n* the “Hill slope”. The Boltzmann relation used to describe voltage dependence of K^+^ current steady-state inactivation is described by the equation:
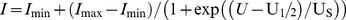
(2)where *I* is current with subscripts “max” and “min” denoting maximum and minimum, *U* is membrane potential, U_½_ membrane potential for half-maximal current and U_S_ a slope factor. The Wilcoxon matched-pairs signed-ranks test was used to statistically evaluate the obtained results, and differences considered significant when p<0.05. Data are presented as mean±S.E.M.

### Quantitative description of currents and modelling of estradiol action

The mathematical description of voltage-gated K^+^ currents follows that of Johansson and Århem [19]. The K^+^ current follows the constant-field equation [20], [21]: 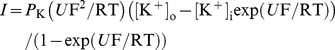
(3)The K^+^ permeability, *P*_K_, depends on membrane potential and time, as a consequence of voltage-dependent, but time-independent rate constants, *α* and *β*, determining the rate of transition between two closed states (C1 and C2) and one open state (O). *P*_K_ is in the model proportional to the number of channels in the open state and is given an arbitrary maximal value since all currents are normalized. The potential dependence of rate constants is given by the following equations: 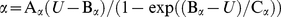
(4)

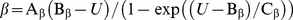
(5) where A_α_ = 0.002 mV^−1^ms^−1^, B_α_ = −50 mV, C_α_ = 4.7 mV, A_β_ = 0.001 mV^−1^ms^−1^, B_β_ = −25 mV and C_β_ = 10 mV as determined by a repeated comparative procedure to obtain a good fit between computed and experimentally recorded currents. The effect of 17-β-estradiol was modelled by the addition of estradiol-bound states and corresponding rate constants as described in the *Results*.

### Solutions and materials

The incubation solution contained (in mM): NaCl 150, KCl 5.0, CaCl_2_ 2.0, HEPES 10, glucose 10, Tris-base 4.9. This solution was oxygenated. The standard extracellular solution contained (in mM): NaCl 137, KCl 5.0, CaCl_2_ 1.0, MgCl_2_ 1.2, HEPES 10, glucose 10, glycine (3 µM), tetrodotoxin (2 µM). pH was adjusted to 7.4 with NaOH. The standard intracellular solution, used for filling of pipettes, contained (in mM): K-gluconate 140, NaCl 3.0, MgCl_2_ 1.2, EGTA 1.0, HEPES 10. pH was adjusted to 7.2 with KOH. The latter solution was filtered (pore size = 0.22 µm), and for back-filling of pipettes (see above), it was supplemented with amphotericin-B (Sigma). Amphotericin-B was dissolved in dimethylsulphoxide (DMSO; 1.2 mg in 20 µl DMSO). The final concentration of DMSO in the intracellular solution was 0.28%. Steroids (17-α- and 17-β-estradiol, obtained from Sigma, and ICI 182,780, obtained from Tocris, Bristol, UK) were first dissolved in 99.5% ethanol, for preparation of solutions containing a final ethanol concentration of 0.2%. In experiments where steroids were used, all solutions were complemented with ethanol to achieve the same final concentration as in the steroid-containing test solution. In addition, for control recordings from two neurons, a solution with DMSO as alternative solvent for 17-β-estradiol was used, with similar results obtained as with ethanol as solvent. (Both solvents were tested in both cells.) The difference in fraction of total K^+^ current blocked by 10 µM 17-β-estradiol, 590–600 ms after a voltage step to +26 mV from −74 mV, was <2% for the two solvents, in both cells tested.) Estradiol bound to bovine serum albumin (β-estradiol 6-(O-carboxymethyl)oxime:BSA) was obtained from Sigma. It was directly dissolved in extracellular solution. The peptide toxins were obtained from Latoxan (Valence, France; α-dendrotoxin) and Alomone (Jerusalem, Israel; r-agitoxin-1 and r-stromatoxin-1) and were similarly dissolved in extracellular solution. Tetraethylammonium chloride (TEA; from Sigma) was added directly to the extracellular solution without compensating for change in osmolarity.

## Results

### 17-β-estradiol reduces K^+^ currents in MPN neurons

**Figure 1:**
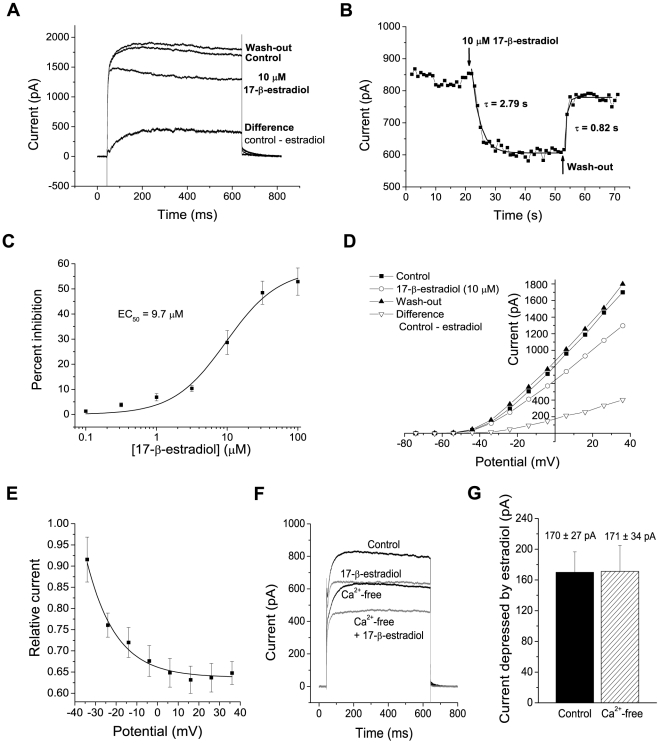
17-β-estradiol rapidly reduces K^**+**^ currents in MPN neurons. A, K^+^ currents evoked by a voltage step from −74 mV to +36 mV, in control solution, after the addition of 10 µM 17-β-estradiol and after wash-out of 17-β-estradiol, as indicated. B, time course of estradiol-induced depression, from the neuron in A. Mean current 190–200 ms after voltage steps to +26 mV from −74 mV. (No leak current subtraction.) 17-β-estradiol was applied as indicated. Superimposed lines show fitted exponentials. C, concentration-response relation for 17-β-estradiol-induced depression of K^+^ currents. Mean currents 590–600 ms after a voltage step to +26 mV from −74 mV. Smooth line is described by Equation 1 with EC_50_ = 9.7 µM, n = 1.2, *Inh*_max_ = 58%. Data from 7 neurons. D, I–V relations for mean current 590–600 ms after a voltage step from −74 mV to the potentials indicated, for one MPN neuron. Current in solutions as indicated. E, relation between the effect of 10 µM 17-β-estradiol (ratio of current in estradiol to that in control solution; mean current 590–600 ms after voltage step from −74 mV) and membrane voltage for 12–13 neurons. Mean±S.E.M. The superimposed line is an exponential function, with *e*-fold change per 14 mV, fitted to the data. F–G, Ca^2+^ independence of estradiol-sensitive current. F, currents evoked by a voltage step from −74 mV to +6 mV, with extracellular solution modified as indicated. Note that estradiol (10 µM) induced a similar depression in the presence and absence of Ca^2+^. G, current depressed (mean current 590–600 ms after a voltage step to +6 mV) by 10 µM 17-β-estradiol added to standard extracellular solution (left bar) and to a solution with Co^2+^ substituted for Ca^2+^ (right bar). The same 9 neurons were used for both conditions. The difference was not significant.

As previously described, MPN neurons generate voltage-gated K^+^ currents in response to voltage steps to potentials>∼−45 mV [22]. Here, such currents were evoked by voltage steps from −74 mV. When 10 µM 17-β-estradiol was added to the extracellular solution, the K^+^ currents were clearly reduced (Fig. 1A). At +26 mV, the current at the end of the voltage step (590–600 ms) was reduced 36 ± 3% (*n* = 13). Further, the time course of current was altered by 17-β-estradiol. There was little or no reduction of current during the first 10 ms after the voltage step, but a higher rate of current decline in the presence of 17-β-estradiol. Thus, the estradiol-sensitive current obtained by subtracting the current in estradiol from that in control showed a roughly exponential time course with time constant 58±6 ms (*n* = 19). The rate of K^+^ current decline, however, increased with the concentration of 17-β-estradiol (see below).

The time course of onset as well as of the reversal of effect, at the start and end of estradiol perfusion respectively (Fig. 1B,) was well fitted by a single exponential with time constant 3.0±0.5 s (*n* = 5) for onset and 1.3±0.2 s (*n* = 5) for reversal. After the exponential onset, the degree of current reduction remained stable for all durations of 17-β-estradiol exposure tested, ranging from 10 s to 10 min, before washout.

Lower concentrations of 17-β-estradiol were not as effective as 10 µM in depressing the K^+^ currents. A concentration-response curve for the depressing effect was generated. The data were well fitted by Equation 1 with an EC_50_ of 9.7 µM, a Hill slope of 1.2 and a maximum inhibition (*Inh*_max_) of 58% (Fig. 1C).

For evaluating possible effects of estradiol on the leak current, current responses to voltage steps from −74 mV to −94 mV were applied. The mean current 190–200 ms after the onset of the step (average responses to 20 steps) was −8.7±0.8 pA in the control solution and −9.1±0.9 pA in 32 µM 17-β-estradiol, when compared in the same 9 neurons. The difference was not significant.

### The effect of 17-β-estradiol on K^+^ currents is voltage-dependent

The K^+^ currents evoked by voltage steps in MPN neurons show an outwardly rectifying relation to the voltage [22] (Fig. 1D). A qualitatively similar I-V relation, but with reduced slope, was seen in the presence of 10 µM 17-β-estradiol. Also the current component sensitive to estradiol showed a qualitatively similar I-V relation (Fig. 1D, lower curve). When the ratio of current in estradiol to that in control solution was plotted *versus* voltage, it was clear that the depressing effect increased with voltage. The relation to voltage was reasonably well fitted by an exponential function, with an *e*-fold change in current reduction per 14 mV (Fig. 1E).

### Ca^2+^-independence

In several preparations, estradiol affects large-conductance Ca^2+^-activated K^+^ channels (see Introduction), which are also voltage dependent. The MPN neurons studied here express several types of Ca^2+^ currents [18] as well as Ca^2+^-activated K^+^ currents [22], [23]. Therefore, we investigated whether the present estradiol-sensitive K^+^ current was dependent on Ca^2+^ influx from the external solution. For this, Ca^2+^ was replaced by Co^2+^ in the extracellular solution, with a resulting reduction in voltage-gated K^+^ current by 16±7% (n = 9; mean current 590–600 ms after a voltage step to +6 mV). However, the current component depressed by 10 µM 17-β-estradiol was similar in the presence and absence of external Ca^2+^, when compared for the same 9 neurons (Fig. 1, F and G). Since removal of external Ca^2+^ completely abolishes the apamin- and bicuculline-sensitive Ca^2+^-dependent K^+^ (SK) current [23] as well as the iberiotoxin- and paxilline-sensitive Ca^2+^-dependent K^+^ (BK) current (Nikolaev, Druzin, Malinina and Johansson, unpublished observations) in MPN neurons, we therefore concluded that the estradiol-sensitive current was mostly a voltage-gated, Ca^2+^-independent K^+^ current.

### Involvement of non-classical receptors and specificity of the depressing effect

**Figure 2:**
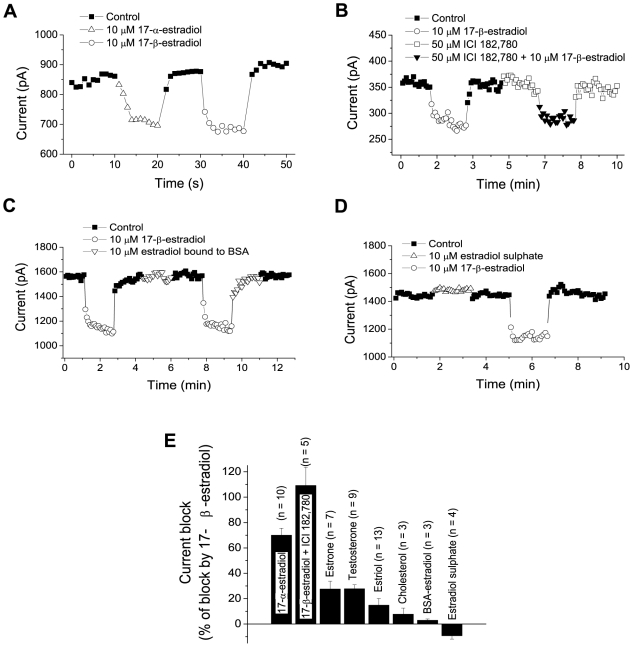
Blocking effects of different steroids on K^**+**^ currents and lack of effect of ICI 182,780. All steroids were applied at a concentration of 10 µM and the estrogen-receptor blocker ICI 182,780 at a concentration of 50 µM. Currents were measured 590–600 ms after voltage steps from −74 mV to +6 mV. A–D, effects of the indicated substances at repetitive voltage steps given to individual neurons. E, summary of blocking effects plotted relative to the blocking effect of 10 µM 17-β-estradiol observed in the same neurons. The number of cells studied for each condition is given within parenthesis. The negative value for estrogen sulphate indicates that this substance caused a slight potentiation of the K^+^ currents.

The rapid time course and the relatively high concentrations needed for estradiol-induced block suggested that a non-classical mechanism of action may be involved. The 17-β-estradiol isomer 17-α-estradiol has been frequently used to separate effects at classical estrogen receptors from those at non-classical receptors (see e.g. [24], [25]) and has also been reported to be inactive at the more recently discovered G protein-coupled estrogen receptor [26]. To obtain information on the receptor types that mediated the presently observed effect on K^+^ currents, we therefore applied 17-α-estradiol. Ten micromolar 17-α-estradiol reduced the voltage-gated K^+^ currents slightly less (15±3%) than did 10 µM 17-β-estradiol (20±3%) when compared in the same 10 neurons (Fig. 2, A and E).

The depressing effect of 17-α-estradiol suggests that G protein-coupled estrogen receptors were not involved but, however, does not completely rule out an involvement of classical estrogen receptors, since also 17-α-estradiol binds those receptors, albeit with lower affinity than 17-β-estradiol [27]. Therefore, we also applied ICI 182,780, which blocks classical estrogen receptors [28] but may function as an agonist at G protein-coupled estrogen receptors [26]. It was clear that ICI 182,780 (50 µM) neither changed the voltage-gated K^+^ currents in the absence of 17-β-estradiol nor prevented the reduction of K^+^ currents caused by 10 µM 17-β- estradiol (Fig. 2, B and E). ICI 182,780 was applied for up to >10 min. (The mean estradiol-induced reduction of current was slightly larger in the presence of ICI 182,780, but the difference from the reduction in the absence of ICI 182,780 was not significant.)

The effect of 17-α-estradiol also suggested that other related molecules may possibly affect the voltage-gated K^+^ channels. We therefore investigated the effects of three related steroids, the other estrogens estriol and estrone and the estradiol precursor testosterone, as well as of cholesterol. Although some depressing effect was noted, all of these substances, applied in a concentration of 10 µM, were considerably less effective in reducing the K^+^ currents when compared with 17-β-estradiol (Fig. 2E). In this respect, the effect of estradiol was relatively specific.

### Effects of membrane impermeant forms of estradiol

To obtain evidence regarding the site of estradiol action, we analysed the effects of two different forms of estradiol that are unable to pass the membrane, 17-β-estradiol covalently bound to bovine serum albumin (BSA) and estradiol sulphate. Neither BSA with a total of 10 µM bound 17-β-estradiol (n = 3) (Fig. 2, C and E) nor 10 µM estradiol sulphate (n = 4) (Fig. 2, D and E) did significantly reduce the K^+^ currents, suggesting that estradiol may have to partition into or pass across the cell membrane to affect the K^+^ channels.

### Mechanism of estradiol action–Subtype of K^+^ current affected

**Figure 3:**
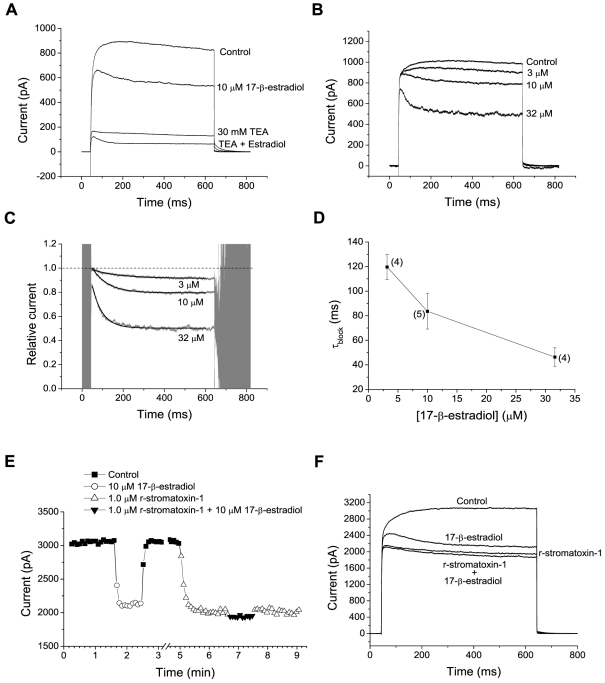
Sensitivity to blockers and concentration-dependent rate of current decline. A, currents (averages of 10 traces) evoked by a voltage step from −74 mV to +6 mV in solutions as indicated. Note that 30 mM TEA blocked a major fraction of the voltage-activated K^+^ current without leaving any significant transient current remaining. Note also that the blocking effects of TEA and 17-β-estradiol were overlapping. B, currents evoked by voltage steps from −74mV to +26 mV, in control solution and in the indicated concentrations of 17-β-estradiol. Note the faster current decline and the larger block with increasing concentration of 17-β-estradiol. C, ratio of current (*grey*) in 17-β-estradiol to that in control solution, to show the time course of current inhibition. The traces were well fitted by exponential functions with time constants 134ms (*top*), 75ms (*middle*) and 49 ms (*bottom*), shown as superimposed black lines. Computed from the traces shown in B. D, dependence of time constant of relative current, as in C, on concentration of 17-β-estradiol. Mean±S.E.M. for the number of neurons indicated. Voltage steps to +26mV from −74 mV. E-F, overlapping block by estradiol and r-stromatoxin-1. E, time-course of block of the K^+^ current (mean current 590–600 ms after a voltage step from −74 to +26 mV) by estradiol, r-stromatoxin-1 and a combination of these substances, as indicated. F, K^+^ currents (averages of 10 traces) evoked by voltage steps from −74 to +26 mV. Concentration of blockers as in E. Note the lack of effect of estradiol in the presence of r-stromatoxin-1. Data in E and F from the same neuron.

As mentioned above, the time course of voltage-gated K^+^ current changed in estradiol, giving an increased rate of current decline during the voltage step. This change in time course was not due to the selective depression of a delayed rectifier current leaving a rapidly inactivating A type, K^+^ current. This was concluded on basis of several observations. First, tetraethylammonium (TEA; 30 mM), which blocks delayed rectifier K^+^ currents but spares A-type K^+^ currents in some preparations, depressed a major fraction (84±3%, n = 6; mean current 590–600 ms after a voltage step from −74 mV to +6 mV) of the voltage-gated K^+^ current recorded, without leaving any significant transient current component. It was thus clear that a major fraction of the current depressed by 10 µM 17-β-estradiol, as well as a major fraction of the current remaining in the presence of 10 µM 17-β-estradiol, was sensitive to TEA (Fig. 3A). However, because TEA may also block some transient K^+^ currents [29], we considered the concentration dependence of the effect of 17-β-estradiol on current time course: If 17-β-estradiol blocks a subtype of current without changing the kinetics, the magnitude of blocked current-but not the time course of current development-should change with estradiol concentration. This, however, was contrary to our observations: The rate, as well as the depth of the depression increased with the concentration of 17-β-estradiol (Fig. 3, B–D).

To further clarify the subtype of K^+^ channel sensitive to 17-β-estradiol, we used several K^+^-channel-selective peptide toxins. Neither α-dendrotoxin (1.0 µM), known to block K_v_ 1.1, K_v_ 1.2 and K_v_ 1.6 channels, nor the K_v_-1.3 blocker r-agitoxin-1 (1.0 µM) affected the K^+^ currents evoked by voltage steps from −74 mV to +26 mV. For both toxins, the remaining current (590–600 ms after the voltage step) was 99±1% (n = 5; data not shown; cf [22]). However, the novel K_v_-2.1, −2.2 and −4.2-blocker r-stromatoxin-1 (1.0 µM) significantly reduced the K^+^ currents (by 51±7%, n = 5; 590–600 ms after a voltage step from −74 mV to +26 mV; Fig. 3, E–F). When 17-β-estradiol (10 µM) and r-stromatoxin-1 (1.0 µM) were applied (separately and in combination) to the same five neurons, the blocking effects were overlapping (Fig. 3, E–F) in all five cells tested. In three of the five cells, the depressing effect of estradiol on the K^+^ currents was abolished by r-stromatoxin-1, and on average, the depressing effect of estradiol was reduced to 26±15% (n = 5) of that in control solution. The overlapping effects suggest a common target of 17-β-estradiol and r-stromatoxin-1.

### Mechanisms of estradiol action on voltage-gated K^+^ channels-altered gating versus pore block

As noted above, the rate, as well as the depth of the block increased with the concentration of 17-β-estradiol (Fig. 3, B–D). Similar findings for quaternary ammonium ions have been explained by an “open channel block” mechanism [30]–[32], a concept that has later been applied to a number of K^+^ channel blockers (for examples, see [33], [34]. We considered if a similar mechanism may explain the time course of K^+^ current in 17-β-estradiol. Alternatively, 17-β-estradiol may alter the intrinsic gating properties of the K^+^ channels.

**Figure 4:**
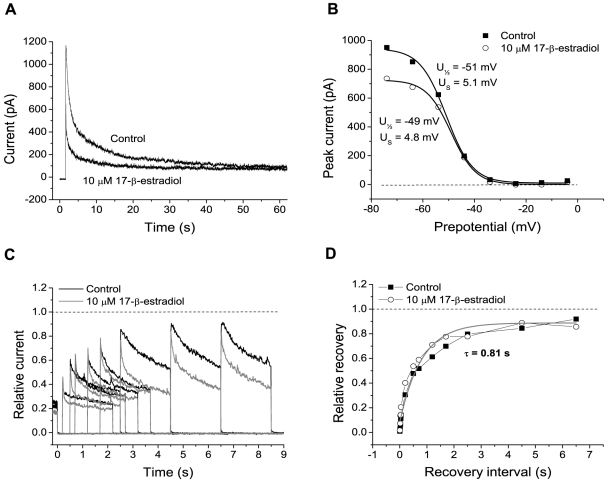
Inactivation of K^+^ currents. A, K^+^ currents evoked by >60 s long voltage steps from −74 mV to +16 mV, in control solution (*upper trace*) and in 10 µM 17-β-estradiol (*lower trace*). Note the slow, but large inactivation in control solution as well as in estradiol. B, voltage dependence of steady-state inactivation. Peak current at +26 mV plotted *versus* the voltage of a 60 s long preceding interval. Control solution (*squares*) and 10 µM 17-β-estradiol (*circles*). Smooth lines are fitted Boltzmann relations (Equation 2) with voltage for half-maximal inactivation (U_½_) and slope factor (U_S_) indicated. A steady non-inactivating current component was subtracted. C, relative recovery from inactivation caused by a 16-s interval at +26 mV (ending at time 0): Superimposed curves represent the currents at +26 mV after varying recovery intervals at −74 mV with and without 17-β-estradiol, as indicated. D, relative recovery for peak currents as in C plotted *versus* the recovery interval at −74 mV. Smooth line is described by a monoexponential function with time constant 0.81 s. The differences between curves for control and estradiol are within the range of trial-to-trial variability for a single condition in these long-duration experiments.

To clarify whether estradiol modifies the gating properties of voltage-gated K^+^ channels in MPN neurons, we had to consider the normal inactivation process of these channels. (Effects on activation *per se* cannot explain the time course of current with a delayed reduction in the presence of estradiol.) Although the K^+^ currents usually show little or no inactivation during the first few hundred milliseconds after an activating voltage step, a major fraction (77±6% after a step from −74 mV to +16 mV; *n* = 8) of the current inactivates during 60-s voltage steps (Fig. 4A, upper trace). The time course of inactivation shows some variability between cells, but is often well described by a sum of two exponentials with time constants 2.4±0.4 s (relative amplitude 0.50±0.06) and 25±10 s (relative amplitude 0.50±0.06; *n* = 8). The steady-state inactivation, as measured by voltage steps to +26 mV after 60-s intervals at different preceding potentials, is well described by a Boltzmann relation (Equation 2, see Materials and Methods) with half-maximal inactivation (U_½_) at −47±3 mV and a slope factor (U_S_) of 7.3±0.7 mV (*n* = 7; Fig. 4B, filled squares), although for these parameters there was a considerable variability between individual cells. The recovery from inactivation (after a 16-s inactivating voltage step to +26 mV) was reasonably well described by one exponential function with time constant 1.37±0.23 s (*n* = 9; Fig. 4, C–D).

In many neurons, long-duration (several seconds) voltage steps, as described above, induced a hump-like outward current component. This component was eliminated when Co^2+^ was substituted for Ca^2+^ in the extracellular solution (not shown), and was therefore attributed to Ca^2+^-dependent currents. Neurons with a prominent Ca^2+^-dependent current component were not included in the present analysis.

Addition of 10 µM 17-β-estradiol, which reduced the current and changed the initial time course, did not significantly change the time course of the slow intrinsic inactivation (Fig. 4A). Neither did estradiol significantly change the voltage for half-maximal steady-state inactivation (U_½_ = −47±3 mV in estradiol; *n* = 7; Fig. 4B), nor the time constant of recovery from inactivation (1.12±0.15 s in estradiol; *n* = 9; Fig. 4, C–D). This suggests that the estradiol-induced depression and the slow intrinsic inactivation were independent phenomena.

**Figure 5:**
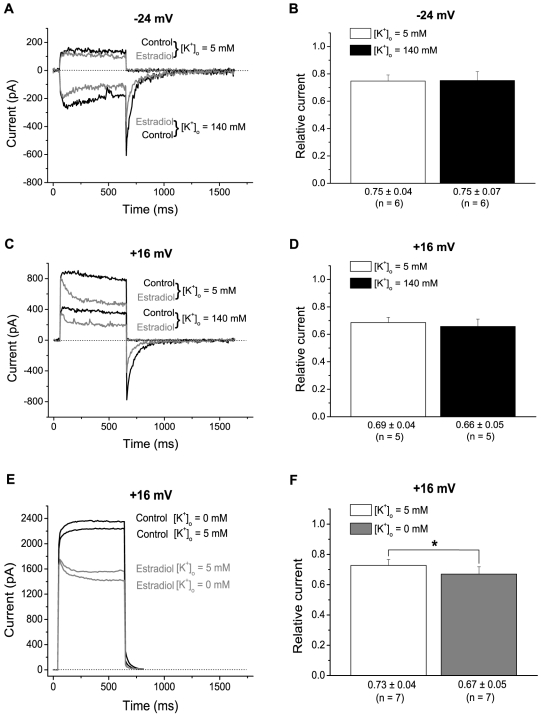
Effects of altered [K^**+**^]_o_ on block by 17-β-estradiol. A, currents recorded at 600-ms voltage steps to −24 mV from −74 mV. At −24 mV currents in standard [K^+^]_o_ of 5 mM are outward (*top traces, control black*) with some reduction caused by 10 µM 17-β-estradiol (*grey*). With a [K^+^]_o_ of 140 mM, currents (*lower traces, control black*) are inward and 10 µM 17-β-estradiol (*grey*) also reduces the current. B, relative current (mean±S.E.M. from 6 cells) in 10 µM 17-β-estradiol, measured 590–600 ms after a voltage step as in A. C, currents as in A but with voltage step to +16 mV. D, relative current (mean±S.E.M. from 5 cells) in 10 µM 17-β-estradiol, measured 590–600 ms after a voltage step as in C. E, currents recorded as in C, but with comparison between standard [K^+^]_o_ of 5 mM and [K^+^]_o_ = 0 mM. F, relative current (mean±S.E.M. from 7 cells) in 10 µM 17-β-estradiol, measured 590–600 ms after a voltage step as in E. Note the slight, but significant difference in blocking effect recorded from the same cells when [K^+^]_o_ = 5 mM and when [K^+^]_o_ = 0 mM.

The lack of effect on slow inactivation suggested that estradiol may possibly block K^+^ channels by plugging the pore. For some other K^+^ channel blockers that plug the pore, the permeant K^+^ ions may clear the pore, giving rise to variations in block with K^+^ concentration and direction of current [30]. Such “knock-out” effects may be expected mainly as a consequence of electrostatic repulsion between K^+^ and positively charged blockers such as the quaternary ammonium ions. Nevertheless, we speculated that to some extent a similar effect may also be seen for estradiol, which although being uncharged have important polar components at each end of the molecule [35]. To test if the estradiol-induced block showed such properties, the block was measured with external K^+^ concentration, [K^+^]_o_, increased to 140 mM (and Na^+^ concentration reduced to keep osmolarity). Then the degree of block was compared with that in the same cells, but with standard [K^+^]_o_ of 5 mM. The degree of block (mean block 590–600 ms after a voltage step from −74 mV) was similar for these two [K^+^]_o_ values, at −24 mV, where current was inward in 140 mM K^+^ but outward in 5 mM K^+^ (Fig. 5, A and B), as well as at +16 mV, where current was outward with either [K^+^]_o_ (Fig. 5, C and D). We reasoned, however, that an effect of external K^+^ may possibly saturate at low [K^+^]_o_. Therefore, we also investigated the effect of reduced [K^+^]_o_. Indeed, when K^+^ was omitted from the external solution, the block (33±5%) was slightly, but significantly, larger than when recorded in the same 7 neurons with standard [K^+^]_o_ of 5 mM (27±4%; Fig. 5, E and F). The latter findings are consistent with the idea that estradiol plugs the pore from the inside and that this process is to a small but significant extent impaired by K^+^ entering from the outside.

### A model of estradiol-induced block of voltage-gated K^+^ channels

**Figure 6:**
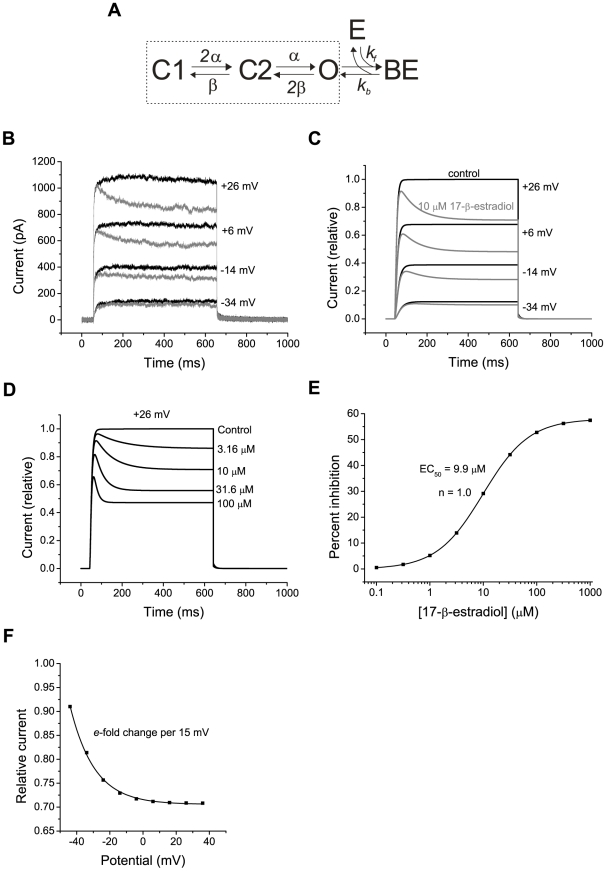
Model of estradiol action on voltage-gated K^**+**^ currents. A, state diagram showing the model with estradiol (E) binding to open channels (O) to form the blocked state with estradiol bound (BE). Dashed box includes the voltage-dependent transitions between closed states (C1 and C2) and the open state. B, experimentally obtained raw data currents from one neuron in control conditions (*black curves*) and in the presence of 10 µM 17-β-estradiol (*grey curves*), for comparison with C. The currents were activated by voltage steps (600 ms) to indicated potentials from a holding potential of −74 mV. C, computed K^+^ currents for control conditions (corresponding to states enclosed by dashed box in A; *black curves*), and for the presence of 10 µM 17-β-estradiol according to the model (*grey curves*). Voltage steps as in B. D, computed currents at different concentrations of 17-β-estradiol, as indicated. Voltage step to +26 mV from −74 mV. E, concentration-response curve for computed currents 600 ms after a voltage step from −74 mV to +26 mV. EC_50_ value and Hill coefficient (*n*) are given in the figure. F, voltage dependence of the block induced by 10 µM 17-β-estradiol (600 ms after a voltage step from −74 mV to indicated potentials) according to the model. The current in 17-β-estradiol is plotted relative to control. The line is a fitted exponential curve, with an *e*-fold change in relative current per 15 mV.

A quantitative description of the voltage-gated K^+^ currents was made with the aim of establishing a simplified model for the blocking action of 17-β-estradiol. For this, we modified the equations used by Johansson and Århem [19] to describe neuronal voltage-gated K^+^ currents. The equivalent state diagram, with two closed states (C1 and C2) and one open state (O), is shown within the dashed boxes in Fig. 6A. The K^+^ permeability was assumed to vary linearly with the number of channels in the open state and the potential-dependent but time-independent rate constants, *α* and *β*, were determined as described in the section Materials and Methods (Equation 4 and 5). The slow intrinsic inactivation of the current was ignored, since this was nearly insignificant during the first 600 ms after activating voltage steps where the effect of estradiol reached steady state. It was also justified by the above finding that the slow inactivation and the estradiol-induced block were independent parallel phenomena. The computed currents, shown in Fig. 6C (black lines), described the experimentally recorded currents (from a “typical” cell: Fig. 6B) well.

For modelling the block induced by 17-β-estradiol, we assumed that estradiol binds to open channels only, in analogy with the open-channel block mechanism for quaternary ammonium ions described above. To match the maximum block observed, it was assumed that half the number of channels constituted an estradiol-sensitive population. The simple model (Fig. 6A) is equivalent to estradiol molecules binding to open channels in a one-to-one manner. Ignoring the activation/deactivation, the rate constant *k_b_* can be derived from the time constant (*τ*) of block when the estradiol concentration is equal to the EC_50_ value: 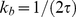
(6)The experimentally recorded time constant of block was about 84 ms at the EC_50_ concentration of 9.7 µM (Fig. 5, B and C). Thus, a *k_b_* of 6.0 s^−1^ is obtained and *k_f_* may be calculated from:
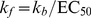
(7)to 0.61 s^−1^ µM^−1^. When these rate constants were used, the computed time course of voltage-gated K^+^ currents in 10 µM estradiol (Fig. 6C, grey lines) was surprisingly similar to that experimentally recorded (Fig. 6B, grey lines). The model successfully described also the concentration-dependence of block, with respect to time course of currents (Fig. 6D) and the steady degree of block (Fig. 6E). Moreover, because the open state communicates with the closed states in a voltage-dependent manner, the relative current in the presence of estradiol was voltage-dependent (Fig. 6F) in a way remarkably similar to that observed experimentally (Fig. 1E).

## Discussion

### Mechanism of action-possible receptor

The present study suggests a new model for rapid effects of 17-β-estradiol on voltage-gated K^+^ channels. It has been previously shown that estradiol may bind to membrane receptors and trigger a fast signal pathway including the production of second messengers [36]. In several reports, however, the effects are observed only after 10–20 minutes (see e.g. [14]). The rapid (within seconds) blocking effect on K^+^ currents in the present study suggests that 17-β-estradiol acts via non-transcriptional mechanisms, possibly directly on the K^+^ channels. The overlapping block by r-stromatoxin and estradiol suggests that a large number of the estradiol-sensitive K^+^ channels may be of K_V_2.1 or K_V_2.2 type. Stromatoxin-sensitive K_V_4.2 channels, which show much quicker inactivation than the presently studied channels and are not sensitive to TEA [29], can most likely be excluded. The effect of 17-α-estradiol and the lack of effect of ICI 182,780 provide support for the idea that “classical” estrogen receptors and G protein-coupled estrogen receptors are not involved. The lack of blocking effect of estradiol sulphate and of estradiol bound to albumin suggests that estradiol acts from the internal side of the cell membrane.

### Mechanism of action-determinants of time course

Two different findings need to be considered with respect to the time course of estradiol action. First, upon perfusion with 17-β-estradiol, the onset of block occurred with a mean time constant of 3.0 s. It seems likely that this time course reflects the change in estradiol concentration at the site of action. We speculate that it partly reflects the passing across the cell membrane and the translocation to the internal cavity of the K^+^ channel. Second, the time course of current activated by each voltage step revealed a relatively rapid decline in the presence of estradiol. We considered two alternative explanations: (i) Estradiol may change the channel gating properties, or (ii) estradiol may require open channels for binding and blocking the pore. One argument, although not conclusive, against the first alternative (i) is that estradiol did not significantly affect other aspects of channel inactivation, but induced the fast current decline in parallel with the slower, intrinsic voltage-dependent inactivation. Thus, estradiol did not simply change the rate of the intrinsic inactivation. Further, the rate and degree of the estradiol-induced decline of current increased with concentration in a manner reminiscent of the block of voltage-gated K^+^ channels by quaternary ammonium ions [30], [31]. For quaternary ammonium ions an “open-channel block” mechanism explains the time course: The drug has access to the binding site only when the channels are open. Further, the block varies with [K^+^]_o_, providing clear evidence for permeant K^+^ ions interacting with the blocker in the pore. Similarly in the present study, the slightly, but significantly, larger block observed in the absence of external K^+^ supports the idea that permeant ions interact with estradiol molecules that plug the pores of open channels from the inside. The quantitative model, discussed below, provided additional support for an open-channel block mechanism.

### Quantitative model of estradiol action

The quantitative model successfully described the time course and voltage dependence of K^+^ currents recorded. Further, under the assumption that estradiol binds to open channels only, the model explained several properties of the observed block: the time course of block, the concentration dependence of time course and the concentration dependence of steady-state block. Remarkably, the voltage dependence of the block was also well described by the model, although no voltage dependence was assumed for the binding or unbinding of estradiol. Rather, the voltage-dependent block is a consequence of binding to open channels only, when the rates of opening and closing are voltage dependent. Thus, our model provides a clear example demonstrating that a voltage-dependent block does not imply voltage-dependent binding (cf. [32]). Notably, a voltage-dependent open-channel block has also been described for another blocker lacking fixed charges [34]. The success of the present model in describing the observed effects may be taken as strong support for the conclusion that estradiol acts as an open-channel blocker of voltage-gated K^+^ channels.

### Comparison with other reported rapid effects of estradiol on voltage-gated K^+^ currents

Two recent studies show rapid effects of 17-β-estradiol on voltage-gated K^+^ currents. In the study by Fatehi et al. [37], the effect appeared within 5 min of application of 17-β-estradiol. However, in contrast to the present work, 17-α-estradiol was ineffective and ICI 182,780 blocked the effect of 17-β-estradiol, suggesting a rapid action via classical estradiol receptors. However, the very high concentration of ICI 182,780 used (1 mM, cf. IC_50_ = 0.29 nM) and the possibility of this drug directly blocking K^+^ channels [38] suggest that the effects may not be due to classical estrogen receptors. The time course of current in 17-β-estradiol was also similar to that in the present study, consistent with open-channel block. In the study by Möller and Netzer [39], it was shown that estradiol reduces K^+^ currents through KCNQ1/KCNE1 channels, which are important regulators of cardiac function, but the mechanism of action was not analyzed. Therefore, we may speculate that 17-β-estradiol acts on voltage-gated K^+^ channels by an open-channel block mechanism in parabrachial neurons and cardiac cells as well as in medial preoptic neurons. It may be noted that a very large number of the known open-K^+^-channel blockers affect cardiac function and include the class III antiarrythmics (for examples, see [33], [40]). Intriguingly, estradiol also has a prominent antiarrythmic effect [41]. The present findings make it tempting to speculate that the antiarrythmic effect is at least partly due to the action of estradiol as an open-channel blocker of voltage-gated K^+^ channels.

### Physiological role of estradiol-block of K^+^ channels in MPN neurons

The physiological role of 17-β-estradiol for MPN neuronal function is largely unknown. Both testosterone and 17-β-estradiol are secreted by the testes into the systemic circulation, and testosterone can be converted by aromatase to 17-β-estradiol in the central nervous system [42]. In addition, estradiol is produced *de novo* from cholesterol in the male brain [8]. Aromatase activity may change within minutes and rapid changes in local estradiol concentration are expected [8], [10]. Since, the preoptic area is particularly rich in aromatase, and since 17-β-estradiol rapidly affects male sexual behaviour [6], which is regulated by the MPN [9], it is tempting to speculate that 17-β-estradiol affects the membrane properties of MPN neurons under physiological conditions.

The high (micromolar) concentrations needed for block are considerably higher than the serum levels of estrogens. However, the serum levels do neither accurately reflect the estradiol concentration in target cells nor the importance for local effects [43]. Massive local production of estradiol may dramatically increase the concentration to micromolar levels in tissue rich in aromatase [44]. The MPN is particularly rich in aromatase [43], suggesting that the local concentration of estradiol may be high. This idea is further supported by findings that although estradiol is known to mediate sexual behaviour by actions in the preoptic area, estradiol concentrations that are toxic to other organs are required when exogenous estrogens are used to activate sexual behavior [1], [10]. Recent findings also show that aromatase inhibitors may rapidly suppress male sexual behaviour in mice. The changes may be restored by injection of estradiol, likely acting via membrane effects, but a high concentration is required (500-µg doses injected in mice; 20 µg being insufficient [7]). (Five hundred µg estradiol is equivalent to a mean concentration in the order of 100 µM in a 25 g mouse.) Thus, it seems rational that while low concentrations of estradiol may mediate mainly the slow effects on sexual behaviour, the rapid effects occurring in a time-scale of minutes may be mediated by locally produced estradiol in high concentrations, in particular in areas such as the MPN where aromatase is highly concentrated [10]. A significant fraction of aromatase is localized to presynaptic terminals and it has been proposed that estradiol may be regarded as a neurotransmitter [45]. Thus, fluctuations in estradiol concentration may be local even at a subcellular scale. In summary, we cannot exclude the possibility that even under physiological conditions, the local concentration of estrogen within the MPN, may be sufficient to affect neuronal K^+^ channels.

### Main conclusion

It is concluded that 17-β-estradiol rapidly blocks delayed rectifier K^+^ channels, likely of K_V_ 2.1 or K_V_ 2.2 type, in MPN neurons and that the properties of block can be explained by an open-channel block mechanism where estradiol binds to K^+^ channels from the inside of the membrane. This implies a new mechanism for steroid action on ion channels that may be relevant also for other preparations.
